# Indicators and Bacterial Diversity of Subclinical Mastitis in Iran's Industrial Cattle Farms

**DOI:** 10.1002/vms3.70951

**Published:** 2026-04-15

**Authors:** Roozbeh Kalantari, Sina Salajegheh Tazerji, Nooriyeh Garaei

**Affiliations:** ^1^ Department of Veterinary Clinical Sciences SR. C., Islamic Azad University Tehran Iran; ^2^ Young Researchers and Elites Club SR. C., Islamic Azad University Tehran Iran; ^3^ AMR – One Health Consortium University of Calgary Calgary Alberta Canada; ^4^ PAHO/WHO Collaborating Centre for Research on Antimicrobial Resistance and the Appropriate Use of Antimicrobials University of Calgary Calgary Alberta Canada

**Keywords:** bacterial diversity, Iran, lactose, milk, SCC, subclinical mastitis

## Abstract

**Background:**

Mastitis is an important and prevalent infectious disease in dairy herds that imposes high economic losses annually to the animal industry. Numerous infectious agents, including bacteria, fungi and viruses, can cause mastitis, with *Staphylococcus* spp. and *Streptococcus* spp. as prevalent causative agents. Mastitis is typically classified as clinical or subclinical, which impacts lactose and somatic cell count (SCC) volumes in milk.

**Material and Method:**

In this cross‐sectional study, 108 Holstein Friesian cows from three industrial farms in Iran were sampled; after exclusion of five samples for culture contamination, a total of 427 milk samples were analysed. Samples with SCC > 200,000 cells/mL were considered positive for subclinical mastitis.

**Results:**

Overall, 238/427 samples (55.7%) exceeded this SCC cut‐off. *Staphylococcus aureus* and *Klebsiella* spp. were the most frequent isolates among culture‐positive samples. Antibiotic susceptibility testing was performed on 11 representative isolates (selected to cover the main species and herds). A significant negative association between milk lactose and log‐SCC was observed (*p* < 0.05).

**Conclusion:**

These results indicate lactose reduction as a useful indirect indicator of subclinical mastitis and document the bacterial profile of industrial Iranian farms.

## Graphical abstract

In this study, the relationship between milk lactose and somatic cell count and the prevalence of bacterial agents of subclinical mastitis was investigated. Klebsiella spp. was identified as the most common pathogen and showed a pattern of antibiotic resistance.

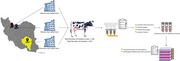

## 1 Introduction

Mastitis is inflammation of the mammary gland characterized by a wide range of physicochemical changes in milk and pathological changes in the gland tissue. The significant changes in milk include colour alteration, the presence of clots and a large number of leucocytes. In many clinical cases, swelling, fever, pain and oedema are found in the mammary gland. However, a large proportion of mastitis is not easily detected by palpation or visual inspection of milk, and such cases are termed subclinical infections. Diagnosis of subclinical mastitis depends largely on indirect tests, which are in turn dependent on biological factors such as elevated somatic cell count (SCC) or electrolyte (sodium or chloride) concentrations in milk (Constable et al. 2016). Recent genomic and molecular studies have shown that mastitis‐causing pathogens possess diverse virulence determinants, host‐adaptation traits and antimicrobial resistance mechanisms that directly influence disease persistence and treatment outcome. For instance, *Staphylococcus aureus* strains associated with bovine mastitis exhibit distinct clonal lineages with specific virulence gene repertoires, biofilm‐forming capacity and host‐specific adaptation mechanisms, which enhance their ability to cause chronic and recurrent infections (Chowdhury et al. 2025). Similar to the last one, another comparative genomic investigation has demonstrated that mastitis‐associated pathogens harbour resistance elements and metabolic pathways allowing survival under milking hygiene stressors and intramammary immune pressure (Chowdhury et al. 2025). The economic burden of these pathogens is further exacerbated by foodborne risks and milk spoilage potential, particularly when coliforms and toxin‐producing organisms are present, as highlighted in recent food safety evaluations (Farabi et al. 2025; Tanni et al. 2025). Taken together, these findings emphasize the need for continuous, region‐specific monitoring of pathogen diversity, virulence profiles and antimicrobial resistance in dairy herds (Asha et al. 2024; Tanni et al. 2025). Furthermore recent surveys and meta‐analyses have shown a wide range of mastitis prevalence depending on region, herd management and diagnostic criteria. For example, a pooled estimate from five regions of Africa estimated subclinical mastitis in cattle farms to be approximately 48.2%. Furthermore, a study in Bavaria, Germany, showed that mastitis pathogens were detected in 19% of Cartier milk samples (Bechtold et al. 2024; Khasapane et al. 2023). In general, mastitis is not dependent on individual cattle breeds. For dairy cows, heritability estimates for clinical mastitis average around 0.05. These low heritability estimates suggest a very slight genetic influence on clinical mastitis, but it is strongly impacted by the environment (Zadoks et al. 2011). In a study conducted on 1341 cows in Arak City, Iran, 18 cows (1.4%) had clinical mastitis, and 467 cows (34.8%) had subclinical mastitis. Out of 561 milk samples from cows with a positive California Mastitis Test (CMT), 29% did not show bacterial growth. From the remaining samples, coagulase‐positive *Staphylococcus* (51%), *Streptococcus agalactiae* (24.62%), coagulase‐negative *Staphylococcus* (9.04%), *S. aureus* (1.76%), *S. dysgalactiae* (0.5%), *S. faecalis* (3.01%), *Actinomyces pyogenes* (4.77%), *Escherichia coli* (1.76%), *Bacillus cereus* (2.01%), *Micrococcus* (1%) and two yeast species (0.5%) were identified (Mahzounieh et al. 2003). A second study in Iran demonstrated that *S. aureus* and *S. agalactiae* were the most common agents causing mastitis on dairy farms in the Shiraz region (Haghkhah et al. 2011). In 2023, B. licheniformis, a Gram‐positive bacterium, was also implicated as a potential cause of mastitis (Costa et al. 2019). As mentioned before, many pathogens can cause mastitis, and S. aureus and S. agalactiae are considered common in many parts of the world. Infected glands of cows in the herd are the usual source of contagious mastitis pathogens, with transmission occurring at milking. In addition, the hands of workers can serve as a source of S. aureus (Madboly et al. 2025). The predominant cow‐to‐cow transmission route is through contaminated teat wipes, milk residue in milking machine cups and milking equipment (Constable et al. 2016). Recent evidence suggests that subclinical mastitis caused by bacterial pathogens commonly present on the teat skin is on the rise. In addition, opportunistic pathogens of the udder can cause intramammary infections by ascending through the mammary canal (Radostits et al. 2007). Environmental mastitis is associated with three main groups of pathogens: coliforms (especially E. coli and Klebsiella), environmental Streptococcus spp and others such as Trueperella pyogenes. The source of these pathogens is the cow environment. Transmission occurs from the environment to cattle and is commonly associated with poor environmental management. Examples include wet bedding, dirty floors, excessive use of water, inadequate udder and udder skin preparation before milking, stocking systems allowing damage to the teat and poor fly control (Rzewuska et al. 2019). The relationship between mastitis development and season varies depending on geographical and climatic conditions. In subtropics and tropics, the incidence of infection may increase the frequency of infection during winter or spring because of increased humidity. In temperate climates, the incidence of mastitis in dairy herds is usually more frequent in summer, which has been attributed to the ambient temperature that facilitates the growth of mastitis pathogens in the bedding (Elghafghuf et al. 2014). Studies on the incidence of mastitis indicate that Blood lactose levels increase in subclinical mastitis cases and more significantly in clinical mastitis. In addition, lower lactose concentrations in cows with intramammary infection are linked to decreased lactose secretion due to increased conversion of plasminogen to plasmin (Leitner et al. 2011). Cows with milk lactose content ≤ 4.553% are reported to have more health problems than those with lactose content ≥ 5.045%. Moreover, subclinical mastitis is genetically correlated with milk lactose, and productive cows are genetically more susceptible to mastitis than low‐producing cows (Costa et al. 2019). Furthermore, a decrease in the milk lactose concentration is associated with coagulase‐negative *Staphylococcus, S. aureus*, coliform bacteria and fungal infections (Kayano et al. 2018).

Subclinical mastitis is, therefore, an important element affecting the quality and quantity of milk produced by dairy cows. Subclinical mastitis is often not diagnosed promptly due to the lack of symptoms. In addition, there is a lack of knowledge regarding the impacts of subclinical mastitis on the levels of milk components on Iranian farms. Understanding these relationships can help with the identification of early, non‐invasive and efficient indicators for subclinical mastitis. Therefore, this study aimed to investigate the bacterial diversity of subclinical mastitis in Holstein Friesian cows in Iran and analyse associations between subclinical mastitis and milk components such as lactose, fat and protein levels. The results of this study can help improve mastitis diagnostics and management in industrial livestock farms in Iran.

## 2 Material and Methods

### 2.1 Study Design and Sample Size Calculation

**FIGURE 1:**
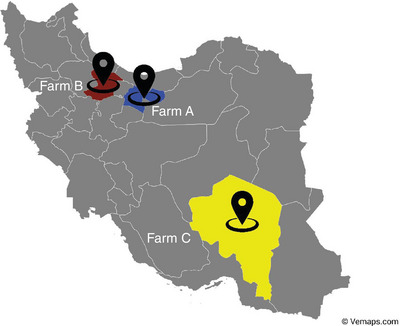
Location map. Map of sampling locations. Three industrial dairy farms located in Tehran (Farm A, 160 milking cows), Qazvin (Farm B, 85 milking cows) and Kerman (Farm C, 145 milking cows) were sampled; distances between farms were at least 100 km. GPS coordinates.

To determine the frequency and bacterial diversity of subclinical mastitis in industrial dairy farms in Iran, three farms were selected in the cities of Tehran, Qazvin and Kerman. Farms were at least 100 km apart (Figure 1).

Farms had different management protocols but with some similarities. Cows were milked every 8 h (e.g., three times daily). After milking, the milking machine was washed with cold water for 10 min, followed by hot water (about 75°C) for 10 min, and finally washed with acidic or alkaline disinfectants prior to reuse. In addition, the milking units were disinfected with caustic soda and weak acids four times a week.

To ensure proportional representation of all participating herds, the sample size was allocated based on the total number of lactating cows in each farm. The three industrial herds included 160 cows in Tehran, 145 cows in Kerman and 85 cows in Qazvin (total: 390 cows). Therefore, the 108 cows enrolled in this study were distributed proportionally among the farms as follows: 44 cows from the Tehran herd, 40 cows from the Kerman herd and 24 cows from the Qazvin herd. This proportional approach ensured that the sample structure reflected the actual population size of each farm and prevented sampling bias.

### 2.2 Sample Collection and Processing

Samples were collected from randomly selected cows during milking, with cows chosen from different milking rows. For sampling, udders were first washed with lukewarm water and then dried with sanitary tissue paper. Thereafter, teats were disinfected with 70% alcohol. The first and second milking streams were discarded, and then ∼15 mL of milk was poured into sampling containers. SCC was first determined indirectly and semi‐quantitatively by the CMT method. For this purpose, equal volumes of milk and CMT reagent were combined and gently mixed until the reaction between the reagent and the cells' DNA resulted in a change in consistency or gel formation. This change was scored on a 0–3 scale. In addition, suspicious samples were analysed by flow cytometry to improve counting accuracy (Li et al. 2015). Based on industry standards and peer literature, samples with SCC > 200,000 cells/mL were classified as indicative of subclinical mastitis and submitted for bacteriological culture.

### 2.3 Bacteriological Culture and Identification

All samples containing more than 200,000 somatic cell units were sent to the ‘Amin Salamat Iranian’ laboratory to determine the diversity and abundance of bacteria. In the laboratory, these samples were first cultured in blood and MacConkey agar and incubated for 48 h. Colonies were Gram‐stained to observe morphology. Presumptive genus classification was accomplished using catalase and oxidase tests, as well as colony aspects and morphology. To identify bacterial species, the IMVIC test, which includes four tests: indole, methyl red, Voges–Proskauer and citrate and the Triple Sugar Iron Agar (TSI) test, was used.

### 2.4 Antimicrobial Susceptibility Testing

A sample of identified isolates, including S. aureus, coagulase‐negative Staphylococcus, E. coli, Klebsiella spp. and Enterococcus spp., was randomly selected for susceptibility testing using the disk diffusion method for the following antimicrobials: ceftriaxone, cloxacillin, enrofloxacin, ampicillin, gentamicin, neomycin, tetracycline, lincomycin, streptomycin and penicillin. The selected panel was dependent on the species to avoid intrinsic resistance. Inhibition zone diameters were used to classify isolates as sensitive, resistant and intermediate following CLSI guidelines (Weinstein 2018).

### 2.5 Statistical Analysis

Data were analysed using SPSS‐25. Descriptive statistics (mean ± SEM) were calculated for milk constituents. One‐way ANOVA was used to assess differences among herds; post‐hoc comparisons were performed with Tukey's test. In addition, logistic regression was used to evaluate the association between SCC (> 200,000 cells/mL) and categorical predictors. Linear regression (using log‐transformed SCC when appropriate) assessed the relationship between lactose concentration and SCC; reported coefficients represent the change in log‐SCC per unit change in lactose. A significance threshold of *p* < 0.05 was used. In addition, the chi‐square test was used to compare the distribution of cell count categories (SCC) between herds. In addition, this test was used to examine whether the distribution of cell frequency was significantly different between herds.

### 2.6 Ethical Aspects

This research was conducted in full compliance with ethical principles and scientific standards. In all stages of the research, especially in the sampling and milking process, efforts were made to avoid any harm or stress to the cows and to observe all animal welfare considerations.

## 3 Results

Of the 432 collected samples, 5 were excluded due to microbial contamination during transportation. Therefore, the final analysis sample size was 427. The composition of milk from three cattle herds in Iran is summarized in Table 1. Among herds, the highest average fat percentage was observed in Kerman, while the lowest was in Tehran. Protein content was highest in Qazvin and lowest in Tehran. Similarly, lactose levels were most significant in Qazvin and lowest in Tehran. The fat‐to‐protein ratio was highest in Kerman and lowest in Qazvin (Table 1).

**TABLE 1:** Comparison of the average milk compounds in three herds from cattle farms in Iran.

Herd	Fat (%)	Protein (%)	Lactose	Fat/protein ratio
Qazvin	1.8^b^	3.15^a^	4.59^a^	0.58^b^
Tehran	1.66^b^	2.75^b^	4.47^a^	0.62^b^
Kerman	2.43^a^	2.83^b^	4.32^b^	0.91^a^
SEM	0.076	0.04	0.038	0.038
*p*‐value	0.0001	0.0001	0.0001	0.0001
Total	± 1.96 0.97	± 2.91 0.51	± 4.46 0.46	± 0.70 0.48

*Note*: Different superscript letters (a and b) within the same row indicate statistically significant differences between herd means based on the post‐hoc multiple comparison test (*p* < 0.05). Means sharing the same letter are not significantly different from each other. The standard error of the mean (SEM).

Table 2 represents the negative and significant regression of SCC from milk lactose levels. It means that the logarithmic number of somatic cells decreases by 0.611 for a one‐unit increase in the milk lactose level.

**TABLE 2:** Regression coefficients and *y*‐intercepts of somatic cell count (SCC) from milk lactose levels.

	Coefficient	SE	*t*‐value	*p*‐value
*y*‐intercept	4.893	0.325	15.04	0.0001
Lactose	−0.611	0.073	−8.420	0.0001

In terms of SCC, the herd from Kerman had significantly higher SCC levels than the others. No significant differences were observed between the Tehran and Qazvin herds (Table 3).

**TABLE 3:** Distribution of cell counts in the examined samples.

Cell count	*N*	*F*
< 200,000	189	44.3
> 200,000	238	55.7
Total	427	

*Note: N* represents the number of samples included in each group. *F* denotes the *F*‐statistic obtained from the one‐way ANOVA test used to evaluate differences among groups. A higher *F* value indicates a greater degree of variation between group means relative to within‐group variation. Statistical significance was determined at *p* < 0.05.

The majority of samples collected had SCC < 200,000 cells/mL (Table 4). Across herds, differences were observed, with the herd in Kerman demonstrating a higher frequency of high SCC samples (Table 5).

**TABLE 4:** Somatic cell count per herd.

Herd	Mean cell count
Qazvin	429.6^b^
Tehran	320.12^b^
Kerman	24.540^a^
SEM	90.29
*p*‐value	048.0

*Note*: Different superscript letters (a and b) within the same row indicate statistically significant differences between herd means based on the post‐hoc multiple comparison test (*p* < 0.05). Means sharing the same letter are not significantly different from each other.

**TABLE 5:** Classification of cell groups in different herds.

	Milk cell count groups (cells/mL)				Total
	> 200,000		< 200,000		
Herd	*N*	*F*	*N*	*F*	
Qazvin	89	0.63	52	0.47	141
Tehran	90	0.61	58	0.49	148
Kerman	59	0.40	89	0.60	148
Chi squared	14.601		*p*‐value		0.001

*Note: N* indicates the number of samples included in each group. *F* represents the *F*‐statistic obtained from the one‐way ANOVA (or GLM) used to test differences among groups.

**FIGURE 2:**
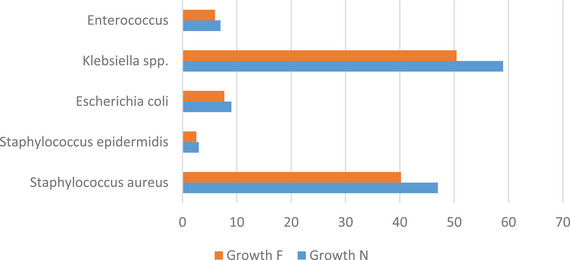
Bacterial frequencies in the cultured milk samples of animals suspected of subclinical mastitis.

*Klebsiella* spp. were isolated in 50.43% of high SCC samples, whereas *S. aureus* were detected in 40.2% (Table 6, Figure 2).

**TABLE 6:** Observed bacterial frequencies in cultured milk samples of animals suspected of subclinical mastitis.

	Growth	
Bacterial species	*N*	*F*
Staphylococcus aureus	47	40.2
Staphylococcus epidermidis	3	2.56
Escherichia coli	9	7.69
Klebsiella spp.	59	50.43
Enterococcus	7	5.98

*Note: N* represents the number of samples included in each analysis. *F* denotes the *F*‐statistic derived from the one‐way ANOVA (or General Linear Model), which was used to assess differences among the evaluated groups. Statistical significance was determined at *p* < 0.05.

Antimicrobial susceptibility testing was performed for 11 isolates: S. aureus (2), coagulase‐negative Staphylococcus (1), E. coli (3), Klebsiella sp. (3) and Enterococcus spp. (2). S. aureus and coagulase‐negative Staphylococcus were susceptible to all tested antimicrobials. E. coli were sensitive to enrofloxacin, gentamicin and tetracycline. Two isolates were resistant to ceftiofur. The three Klebsiella spp. isolates were susceptible to ceftiofur, enrofloxacin and gentamicin. The Enterococcus spp. Isolate was susceptible to enrofloxacin, ampicillin and penicillin (Table 7).

**TABLE 7:** Antibiogram results for bacterial samples isolated from the milk of cattle farms in Iran.

			*Ceftiofur*	*Cloxacillin*	*Enrofloxacin*	*Ampicillin*	*Gentamicin*	*Neomycin*	*Tetracycline*	*Lincomycin*	*Streptomycin*	*Penicillin*
*Staphylococcus aureus*	47	*r*	2	5	2	2	0	4	3	4	5	3
		*s*	45	40	45	45	47	43	44	43	42	44
Coagulase‐negative *Staphylococcus*	3	*r*	0	0	0	0	0	0	0	0	0	0
		*s*	3	3	3	3	3	3	3	3	3	3
*Escherichia coli*	9	*r*	9	NT	1	9	0	9	4	NT	9	9
		*s*	0	NT	8	0	9	0	5	NT	0	0
*Klebsiella*	59	*r*	59	NT	36	NT	0	21	55	NT	25	24
		*s*	0	NT	23	NT	59	38	4	NT	34	35
*Enterococcus*	7	*r*	NT	NT	1	0	NT	NT	1	NT	NT	0
		*s*	NT	NT	6	7	NT	NT	6	NT	NT	7

Abbreviations: NT, not tested due to intrinsic resistance; *s*, sensitive; *r*, resistant.

## 4 Discussion

The results of this study showed that composite milk quality indices, particularly lactose concentration, can be used as accurate and reliable markers for the detection of subclinical mastitis in industrial farms. Regression analysis showed that increased lactose was significantly associated with decreased SCC; this finding is consistent with the known physiological mechanisms of mastitis, where inflammation of the mammary gland leads to dysfunction of epithelial cells and reduced lactose synthesis (Costa et al. 2019; Petrovski et al. 2011). This relationship has been confirmed in global studies, and lactose is increasingly being introduced as a non‐invasive biomarker in herd monitoring programs (Petrovski et al. 2011).

Differences in SCC between herds may reflect health status, litter management, ventilation and milking protocols. Previous studies have shown that factors such as heat stress, environmental humidity and the quality of pre‐milking management can significantly vary SCC (Zadoks et al. 2011). Our findings also support the view that management differences may have a greater impact on the incidence of subclinical mastitis than genetic or nutritional differences. Examination of bacterial abundance revealed that *Klebsiella* spp. was the most common pathogen isolated, with a prevalence of 50.43% and played a dominant role in the incidence of subclinical mastitis in the studied herds. This finding is consistent with the reported pattern for environmental pathogens, which typically thrive in wet bedding, faecal contamination and non‐sterile milking equipment (Zadoks et al. 2011). Furthermore, recent molecular studies have shown that *Klebsiella pneumoniae* strains, in addition to their ability to cause severe infections, often carry virulence genes and transferable antibiotic resistance, which increases the risk of developing MDR/XDR strains (Chowdhury et al. 2025). The high prevalence of *Klebsiella* in the present study, in line with recent reports, suggests that environmental management interventions, including improved litter quality, moisture control and enhanced pre‐milking hygiene, are essential to limit the risk of infection.

*S. aureus* was the second most common pathogen in the study (40.2%). This prevalence is consistent with findings reported in dairy cattle populations in New Zealand and Iran, as well as recent international studies (Campos et al. 2022; Petrovski et al. 2011). Given that *S. aureus* is mainly spread through cow‐to‐cow transmission during the milking process, the role of proper management of milking equipment, udder disinfection and identification and removal of chronically infected cows is prominent in its control. *E. coli* bacteria contributed less to the infections (7.69%), but their antibiotic resistance pattern is clinically important. The present results showed that some *E. coli* isolates were resistant to some drugs, while remaining susceptible to enrofloxacin, gentamicin and tetracycline. This pattern is consistent with previous reports and emphasizes the need for antibiotic selection based on susceptibility testing (Zadoks et al. 2011). Antibiogram results for *Klebsiella* spp. Showed that although many isolates showed acceptable susceptibility, recent reports have confirmed the presence of resistance genes in strains of this genus, which reinforces the importance of continuous monitoring and prudent use of antibiotics (Chowdhury et al. 2025). Recent studies also emphasize the importance of determining the resistance pattern of mastitis pathogens, especially in conditions where transmission of resistance genes is increased (Chowdhury et al. 2025; Tanni et al. 2025).

Overall, the results of this study suggest that the combination of quantitative monitoring of milk components such as lactose, SCC assessment and accurate pathogen identification can provide a comprehensive picture of the udder health status in industrial herds. Based on our findings, the control of environmental pathogens such as *Klebsiella* should be a top management priority, while monitoring antibiotic resistance is essential for selecting appropriate treatment. Future studies using complementary molecular methods investigate resistance patterns, virulence genes and possible environmental and cow‐to‐cow transmission routes of these bacteria.

## 5 Conclusion

According to our comparative evaluation of milk lactose levels and SCC in the studied herds, it can be concluded that the milk lactose concentration is significantly related to SCC. Therefore, lactose can be considered an indirect factor indicative of subclinical mastitis, which the veterinarian and the farm manager can use to detect the mastitis condition in the farm. According to the evaluation, all the extracted bacteria were sensitive to ceftiofur.

Dairy companies and milk collection centres reporting protein, fat and SCC in the analysis of milk delivered by livestock farmers are recommended to clean the udder with dry paper towels or nano‐dry towels before milking.

### 5.1 Limitations

This study has several limitations. Firstly, molecular confirmation of isolates (e.g., PCR or sequencing) was not performed; therefore, species identification was based on biochemical tests and culture, which might misidentify some strains. Secondly, potential contamination during sampling or transport cannot be excluded entirely despite aseptic techniques; to mitigate this, we excluded samples with evident contamination. Third, only a subset of isolates (*n* = 11) underwent antimicrobial susceptibility testing, which limits the generalizability of resistance findings. Future studies should include molecular typing and a larger, systematic AST panel to provide more robust resistance surveillance.

## Author Contributions

**Roozbeh Kalantari**: conceptualization, methodology, data curation, formal analysis, validation, investigation, writing – original draft, writing – review and editing, visualization, project administration, resources, software. **Sina Salajegheh Tazerji**: conceptualization, investigation, writing – original draft, writing – review and editing, visualization, validation, methodology, software, formal analysis, project administration, resources, data curation. **Nooriyeh Garaei**: writing – original draft, investigation, conceptualization, methodology, validation, visualization, writing – review and editing, project administration, formal analysis, software, data curation, resources.

## Funding

The authors have nothing to report.

## Disclosure

The authors have nothing to report.

## Conflicts of Interest

The authors declare no conflicts of interest.

## Data Availability

Supporting data for this study are available upon request from the corresponding author.
